# Effects of Embryo Production Method and Culture Medium on Embryonic Development in Red‐Rumped Agouti

**DOI:** 10.1002/cbin.70080

**Published:** 2025-09-11

**Authors:** Lhara Ricarliany Medeiros de Oliveira, Leonardo Vitorino Costa de Aquino, Luana Grasiele Pereira Bezerra, Moacir Franco de Oliveira, Alexandre Rodrigues Silva, Alexsandra Fernandes Pereira

**Affiliations:** ^1^ Laboratory of Animal Biotechnology Federal Rural University of Semi‐Arid Mossoró Brazil; ^2^ Laboratory of Animal Germplasm Conservation Federal Rural University of Semi‐Arid Mossoró Brazil; ^3^ Laboratory of Applied Animal Morphophysiology Federal Rural University of Semi‐Arid Mossoró Brazil

**Keywords:** artificial oocyte activation, blastocyst development, caviid rodent, early cleavage dynamics, in vitro fertilization, oxidative stress

## Abstract

Advances in Assisted Reproductive Technologies (ARTs) help overcome reproductive barriers. However, a comprehensive understanding of embryonic development is crucial for their success. *In vitro* fertilization (IVF) and artificial oocyte activation (AOA) are embryo production methods commonly used. Previous studies have reported low developmental success with these methods, possibly due to the culture medium used. Therefore, we aimed to optimize ARTs in red‐rumped agouti by evaluating the influence of production method and culture medium on embryonic development. Oocytes were matured in vitro and divided into two groups: IVF with capacited spermatozoa or AOA with strontium chloride and cytochalasin B. Presumed zygotes were cultured in either KSOM or SOF medium after a 6‐h incubation. Morphology, oocyte‐sperm interaction, developmental kinetics, and oxidative stress levels were assessed. IVF‐derived structures exhibited a higher rate of normal morphology compared to AOA‐derived structures (*p* < 0.05). Embryo kinetics analysis showed that AOA‐KSOM and IVF‐KSOM groups had a higher number of cleaved structures than the SOF groups (*p* < 0.05) on Day 2. On Day 5, the IVF‐KSOM group presented the highest percentage of cleavage/total zygotes and the highest percentage of structures with eight or more cells (*p* < 0.05). Morula formation was significantly higher in IVF‐KSOM and AOA‐KSOM (*p* < 0.05), highlighting KSOM's effectiveness. Notably, only the IVF‐KSOM group produced blastocyst (*p* < 0.05). Oxidative stress assessment showed no differences between groups (*p* > 0.05). These findings confirm that IVF‐KSOM is the most effective methodology for developing red‐rumped agouti embryos, offering valuable insights into the reproductive biology of this species and aiding in the refinement of protocols for closely related rodents.

**Abbreviations**
ARTs: Assisted reproductive technologies
AOA: Artificial oocyte activation
BSA: Bovine serum albumin
CASA: Computer‐assisted sperm analysis
ECAU: Ethics committee of animal use
FBS: Fetal bovine serum
H_2_DCFDA: 2′,7′‐dichlorodihydrofluorescein diacetate
HOST: Hypoosmotic swelling test
ICMBio: Chico Mendes institute for biodiversity conservation
KSOM: Potassium‐enriched simplex optimized medium
IVD: *In vitro* development
IVEP: *In vitro* embryo production
IVF: *In vitro* fertilization
IVM: *In vitro* maturation
MCM: Minimum capacitation medium
PBS: Phosphate‐buffered saline
ROS: Reactive oxygen species
SOF: Synthetic oviductal fluid

## 1 Introduction

Advances in Assisted Reproductive Technologies (ARTs) have become crucial for the conservation of wildlife species, such as the red‐rumped agouti (*Dasyprocta leporina* Linnaeus 1758), a seed‐scattering rodent of great importance on the South American continent (Hadler et al. 2016). The ARTs offer innovative solutions to overcome reproductive barriers, enabling the preservation of genetic diversity and facilitating the breeding of species in captivity (Comizzoli and Holt 2019). These technologies allow the collection and storage of gametes, as well as the development of embryos under controlled conditions, thereby contributing to species recovery efforts (Bolton et al. 2022).

However, it is essential to thoroughly understand the embryonic development of these species since the ability to sustain embryos without compromising their developmental potential is crucial for the success of these ARTs (Gardner 2021). *In vitro* systems are extensively used to elucidate various aspects of reproductive physiology (Besenfelder and Havlicek 2023) due to the challenges associated with observing these phenomena in vivo, including gamete interactions (Cañón‐Beltrán et al. 2023), alterations in gene expression (Zhao et al. 2021), and pre‐implantation embryo kinetics (Cañón‐Beltrán et al. 2024). Among the most frequently employed methods for embryo production in such studies are in vitro fertilization (IVF) and artificial oocyte activation (AOA).

The AOA facilitates the study of early developmental processes due to its resemblance to fertilized embryos in gene transcription patterns up to the blastocyst stage, resulting in initial cleavages that are identical to those of fertilized zygotes (Galis and Van Alphen 2020). Additionally, AOA requires a smaller number of animals, as only the mature oocyte is activated without requiring sperm, making it a valuable approach for studies in wild species (Paffoni et al. 2008). However, embryos generated through AOA may exhibit a higher rate of apoptosis and are incapable of developing to term (Daughtry and Mitalipov 2014), highlighting the need also to investigate embryos with a complete genetic display, as obtained through IVF.

Embryo production via IVF provides deeper insights into gamete interactions, as its application needs optimal sperm selection and capacitation, as well as precise determination of the appropriate time and environment for co‐incubation and development (Cañón‐Beltrán et al. 2024). Furthermore, the inclusion of male genetic material enables the expression of Y chromosome‐linked genes, which may influence developmental outcomes, particularly in the later stages of the pre‐implantation period (Latham et al. 2002). Therefore, it is possible to assess distinct physiological responses during the embryonic development of the red‐rumped agouti by comparing these two methods of embryo production.

Additionally, research conducted by Praxedes et al. (2023) on red‐rumped agouti demonstrated that the AOA protocol developed for this species was ineffective, with a developmental success rate of only 5%–9%, as embryos failed to progress beyond the morula stage. This limited efficiency may be attributed to the in vitro development (IVD) medium used, the synthetic oviductal fluid (SOF), since potassium‐enriched simplex optimized medium (KSOM) is commonly employed for rodent embryos (Cañón‐Beltrán et al. 2021; Omid Banafshi et al. 2021). The differences in protein and energy composition between these media may influence embryonic development, as each is designed to meet the specific requirements of the species (Banrezes et al. 2025).

Given this context, the study aimed to evaluate the in vitro developmental potential of red‐rumped agouti embryos produced by two different methods, IVF and AOA, and to assess the effects of IVD media on these structures. These findings will contribute to a deeper understanding of the reproductive physiology of these species and facilitate the optimization of ARTs, ultimately supporting conservation efforts for red‐rumped agoutis.

## 2 Materials and Methods

Unless stated otherwise, the chemicals were obtained from Sigma Chemical Co. (St. Louis, MO, USA).

### 2.1 Experimental Design

**Figure 1:**
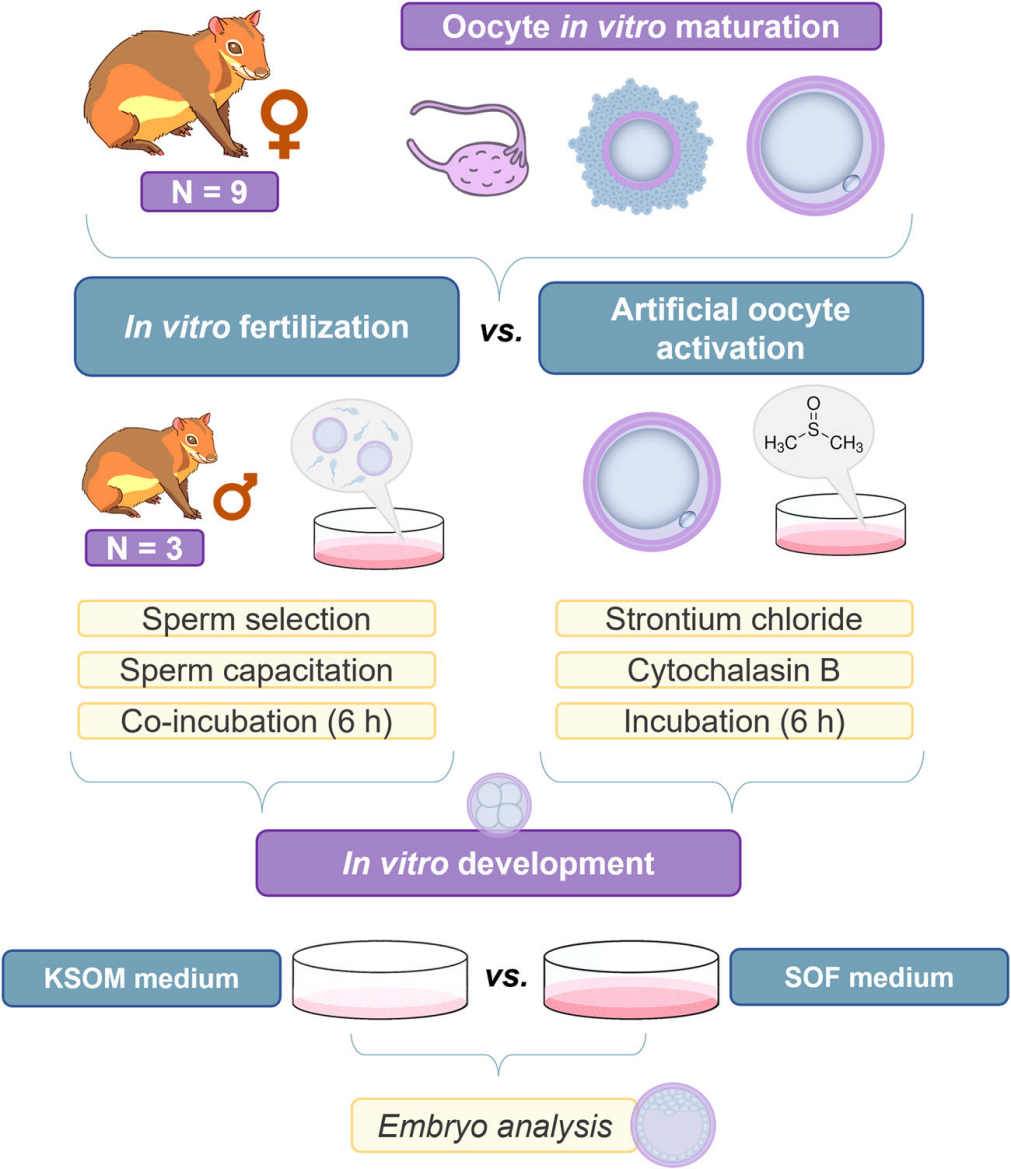
Experimental design: comparative analysis of two in vitro embryo production methods (in vitro fertilization vs. artificial oocyte activation) and optimization of the best in vitro development media in the red‐rumped agouti.

This study was divided into two comparisons to assess the in vitro embryo production (IVEP) potential of the red‐rumped agouti (Figure 1). The first part aimed to compare the efficiency of two methods—IVF and AOA—in supporting the embryonic development of red‐rumped agouti, while the second part evaluated the impact of different IVD media compositions (KSOM vs. SOF) on the structures generated.

Oocytes were subjected to in vitro maturation (IVM) and assessed for nuclear maturation based on the extrusion of the first polar body. Moreover, sperm used for IVF were evaluated for viability through analyses of sperm kinetics, membrane functionality and integrity, mitochondrial activity, DNA damage levels, and morphology. Post‐IVF, resulting structures were examined for oocyte‐sperm interaction, while all presumptive zygotes from both methods were assessed for morphology, embryonic kinetics, and oxidative stress following IVD.

### 2.2 Bioethics and Animals

All procedures were conducted in strict accordance with the guidelines established by the Ethics Committee on Animal Use (ECAU) of the Federal Rural University of Semi‐Arid (Approval no. 20/2021) and the Chico Mendes Institute for Biodiversity Conservation (ICMBio, Approval no. 76655‐1). The red‐rumped agoutis and guinea pigs were housed at the Center for Multiplication of Wild Animals (UFERSA, Brazil) in individual or paired enclosures (2.0 × 1.5 × 1.0 m) made of masonry and equipped with a slatted floor, nest boxes, and environmental enrichment such as branches and foliage. They had ad libitum access to clean drinking water and a standardized commercial rabbit food supplemented daily with seasonal fruits and vegetables. Animals were maintained under a natural 12‐h light/dark cycle, with ambient temperature ranging from 25°C to 30°C, ensuring conditions similar to their natural habitat.

For spermatozoa recovery, three adult male red‐rumped agouti specimens (one male per replicate) were premedicated with an intramuscular injection of ketamine (15 mg/kg; Ketalar, Pfizer, São Paulo, Brazil) combined with xylazine (1 mg/kg; Rompun, Bayer, São Paulo, Brazil). After a 15‐min interval, anesthesia was induced via intramuscular administration of sodium thiopental (50 mg/kg; Thiopentax, Cristalia, São Paulo, Brazil). Euthanasia was subsequently performed through intravenous administration of potassium chloride (1 mL/kg; Equiplex, Goiânia, Goiás, Brazil), following the protocol described by Castelo et al. (2015). For oocyte recovery, nine adult female red‐rumped agoutis (three females/replicate) were anesthetized and euthanized with the same protocol as the male red‐rumped agoutis.

### 2.3 Sperm Preparations and Analysis for IVF

The testes‐vas deferens‐epididymis complex was retrieved immediately following euthanasia and immediately transported to the laboratory in a pre‐warmed saline solution (37°C, 0.15 M NaCl). The cauda epididymis region was carefully dissected, and epididymal spermatozoa were collected via retrograde flushing using 1.0 mL of saline solution (0.15 M NaCl) (Castelo et al. 2015). The sperm samples were maintained in a water bath at 37°C, while preliminary assessments of their appearance, pH, vigor, and concentration were conducted.

The spermatozoa concentration was adjusted to 100 × 10⁶ sperm/mL using a minimum capacitation medium (MCM) composed of 105.8 mM NaCl, 25 mM NaHCO₃, 5.56 mM glucose, 21.6 mM sodium lactate, 25 mM HEPES, 0.25 mM sodium pyruvate, 10 µg/mL phenol red, and 1% antibiotic‐antimycotic solution. A centrifugation protocol was employed to isolate the most viable spermatozoa. A 1:1 mixture of spermatozoa and MCM was transferred to a 15 mL plastic tube and subjected to two centrifugation cycles (300× g for 3 min at room temperature). After each centrifugation, the supernatant was carefully discarded, and the resulting pellet was resuspended for IVF (Oliveira et al. 2025a).

Furthermore, sperm capacitation was performed concurrently with the 6‐h IVF procedure. The capacitation agents used included 4 mg/mL bovine serum albumin (BSA) and 2 mM calcium chloride (CaCl₂), both of which were incorporated into the IVF medium in droplets (50 µL) overlaid with mineral oil (Oliveira et al 2025c). The procedure was performed under controlled environmental conditions at 38.5°C and 6.5% CO_2_.

#### 2.3.1 Sperm Analysis

The epididymal spermatozoa were evaluated using a computer‐assisted sperm analysis (CASA) system (IVOS 7.4 G; Hamilton‐Thorne Research, MA, USA) with parameters previously established for red‐rumped agouti by Castelo et al. (2015). The settings included a temperature of 37°C, a straightness threshold of 30%, a minimum contrast of 45, a low‐velocity average pathway (VAP) cutoff of 10 μm/s, and a medium VAP cutoff of 30 μm/s. Five independent and nonconsecutive microscopic fields were systematically examined. The following kinetic parameters were assessed: total motility (TM, %), progressive motility (PM, %), average path velocity (VAP, μm/s), straight‐line velocity (VSL, μm/s), curvilinear velocity (VCL, μm/s), amplitude of lateral head displacement (ALH, μm), beat cross frequency (BCF, Hz), straightness (STR, %), and linearity (LIN, %). The sperm population was further categorized into four distinct groups: rapid, medium, slow, and static (%).

The sperm membrane functionality was analyzed using the hypoosmotic swelling test (HOST), which involves a solution consisting of distilled water (0 mOsm/L) with a sodium citrate and fructose solution (50 mOsm/L), as defined for the red‐rumped agouti by Dantas et al. (2022). Spermatozoa samples were combined with the hypoosmotic solution and incubated in a dry bath at 37°C for 40 min. The samples were assessed under a phase‐contrast light microscope at 400× magnification. The sperm were identified as possessing a functionally intact membrane when displaying a swollen and coiled tail.

For the membrane integrity and mitochondria functionality, a fluorescence microscopy analysis was performed combining 40 µg/mL Hoechst 33342 (Molecular Probes, Eugene, OR, USA) at 37°C for 5 min, followed by 0.5 mg/mL propidium iodide (Thermo Fisher Scientific, Whaltam, MA, USA) and 500 nM CMXRos (Mito Tracker Red®, F‐7512, Molecular Probes, Eugene, OR, US) for 8 min. Samples were evaluated using fluorescence microscopy (Olympus BX51TF, Tokyo, Japan) at 400× magnification. Spermatozoa exhibiting a blue‐stained head and a red‐glowing midpiece were classified as possessing an intact plasma membrane and normal mitochondrial function (Santos et al. 2023).

The samples were prepared as smears and allowed to air‐dry to detect the level of DNA damage in sperm samples. The slides were subsequently fixed in Carnoy's solution for 3 h and dried at room temperature. They were then incubated for 25 min in a buffer solution consisting of 15 mM Na₂HPO₄ and 80 mM citric acid (pH 2.5) at 75°C. The smears were stained with acridine orange (0.2 mg/mL) for 10 s, rinsed with distilled water, and covered with a coverslip. A total of 100 cells were examined using fluorescence microscopy (400×; Olympus BX51TF, Tokyo, Japan). Spermatozoa with normal (double‐stranded) DNA exhibited a green, fluorescent emission, while those with denatured or single‐stranded DNA displayed yellow, orange, or red fluorescence, indicating progressively higher levels of DNA damage (Tomov et al. 2020).

For morphological assessment, an aliquot of the sperm samples was fixed and stained using a formaldehyde‐Bengal rose solution (Cromato®). The samples were then examined under a light microscope at 1000× magnification, with 100 cells evaluated per slide. The spermatozoa were categorized into two groups: normal or abnormal morphology. Abnormalities were further classified as defects in the head, midpiece, or tail regions (Silva et al. 2011).

### 2.4 Oocyte IVM and Evaluations

The IVM of red‐rumped agouti oocytes was performed following the protocol previously described for this species by Praxedes et al. (2023). The ovaries were collected, stored in pre‐warmed saline solution with antibiotics (0.15 M NaCl, 0.05 mg/mL penicillin, 37°C), and immediately transported to the laboratory. Under stereomicroscopic guidance, all follicles were sliced using scalpel blades to retrieve immature *cumulus*‐oocyte complexes (COCs). COCs with at least one layer of compacted *cumulus* cells and homologous or heterologous ooplasm were selected for IVM. The structures were matured in drops (100 µL, 10–20 oocytes) covered with mineral oil in a controlled atmosphere at 38.5°C and 6.5% CO_2_ for 24 h.

The oocyte maturation medium was composed of TCM199 supplemented with 2.2 g/L sodium bicarbonate, 25 mM HEPES, 0.23 mM sodium pyruvate, 100 µm cysteamine, 10 ng/mL epidermal growth factor (EGF), 10 mIU/mL follicle‐stimulating hormone (FSH; Folltropin®, Vetoquinol, Brazil), 10% fetal bovine serum (FBS), and 1% antibiotic–antimycotic solution with a 7.2 pH (Praxedes et al. 2023).

Following the 24‐h maturation, COCs were examined under a stereomicroscope to evaluate the extent of *cumulus* cell expansion. Structures demonstrating pronounced expansion and mucification were classified as mature. After the removal of excess *cumulus* cells through pipetting, the presence or absence of the first polar body (1PB) was documented using an inverted microscope (Leipzig IMx 400, PhoenixOptics, Germany). Oocytes exhibiting clear evidence of first polar body extrusion were identified as mature (Praxedes et al. 2023).

### 2.5 Red‐Rumped Agouti Embryo Production: IVF versus AOA

For the IVF embryo production, 50 µL droplets were prepared using Tyrode's albumin lactate pyruvate medium (114 mM NaCl, 3.2 mM KCl, 0.35 mM NaH₂PO₄, 10 mM Na lactate, 25 mM NaHCO₃, 2 mM CaCl₂, 0.50 mM MgSO₄, 10 mM HEPES, 4 mg/mL BSA, 0.11 mM sodium pyruvate, 10 µg/mL phenol red, and 1% antibiotic–antimycotic solution, TALP) (Rath et al. 1999), which were then covered with mineral oil and balanced at 38.5°C in 6.5% CO₂ for approximately 30 min. A final sperm concentration of 1.0 × 10⁶ sperm/mL was added to 10–20 intact *cumulus*‐enclosed matured oocytes per drop. The IVF procedure was performed for 6 h at 38.5°C in 6.5% CO₂. Subsequently, the resulting structures were gently washed and pipetted to remove residual spermatozoa and *cumulus* cells before proceeding with in vitro development (Oliveira et al. 2025b).

The protocol described by Praxedes et al. (2023) for the AOA of red‐rumped agoutis was used. Denuded mature oocytes were activated in calcium‐free TALP medium (114 mM NaCl, 3.2 mM KCl, 0.35 mM NaH₂PO₄, 10 mM Na lactate, 25 mM NaHCO₃, 0.5 mM MgSO₄, 10 mM HEPES, 4 mg/mL BSA, 0.11 mM sodium pyruvate, 10 µg/mL phenol red, and 1% antibiotic–antimycotic solution) with 10 mM strontium chloride (SrCl₂) and 5 μg/mL cytochalasin B for 6 h at 38.5°C and 6.5% CO₂. Subsequently, the activated structures were washed for preparation for IVD.

### 2.6 In Vitro Embryo Development: Ksom versus Sof

Structures derived from IVF and AOA were divided between two IVD media drops (50 μL) with either Potassium Simplex Optimized Medium (KSOM) or Synthetic Oviduct Fluid medium (SOF). The KSOM composition was 95 mM NaCl, 2.5 mM KCl, 0.35 mM KH_2_PO_4_, 10 mM Na lactate, 0.2 mM glucose, 25 mM NaHCO_3_, 1.71 mM CaCl_2_, 0.20 mM MgSO_4_, 21 mM HEPES, 0.01 mM phenol red, 0.2 mM sodium pyruvate, 0.01 mM EDTA, 1 mM l‐glutamine, 15 mM BSA, 1% antibiotic–antimycotic solution, 1% essential amino acid solution, 0.5% non‐essential amino acid solution, and 10% FBS (Lawitts and Biggers 1993). SOF was composed of 0.2 mM sodium pyruvate, 0.2 mL l‐glutamine, 0.34 mM citrate sodium, 2.8 mM myo‐inositol, 2% essential amino acid solution, 1% non‐essential amino acid solution, 1% antibiotic–antimycotic solution, 5 mg/mL BSA, and 2.5% fetal bovine serum (Santos et al. 2023). IVD was conducted at a controlled atmosphere at 38.5°C and 6.5% CO_2_. After 48 h of culture (Day 2, D2), 50% of the media was changed. The total culture time was 120 h (Day 5, D5) (Praxedes et al. 2023).

#### 2.6.1 Zygotes Evaluation

After the 6 h incubation, each presumptive zygote was analyzed under an inverted microscope (Leipzig IMx 400, PhoenixOptics, Germany). The zygotes were carefully manipulated to allow a comprehensive evaluation of their morphological traits and the degree of cytoplasmic fragmentation. Zygotes displaying favorable characteristics were classified as normal, following the criteria established by Hesters et al. (2008).

The normal zygotes were cultured for 5 days (120 h) to evaluate their developmental kinetics using an inverted microscope (Leipzig IMx 400, PhoenixOptics, Germany). Cell cleavage was observed on D2 and D5 and classified into three categories: two cells, three to seven cells, or eight or more cells. Furthermore, the percentage of morula formation was determined on D5, along with the ratio of morulae and blastocysts to the total number of cleaved structures (Praxedes et al. 2023). The expanded blastocyst was incubated with Hoechst 33342 for 15 min and then washed in phosphate‐buffered saline/bovine serum albumin. Then, a digital image of the blastocyst was captured by fluorescence microscopy at 40× (370 nm).

The non‐cleaved cells on D2 were analyzed for oocyte‐sperm interaction by staining with Hoechst 33342 (10 µg/mL, 30 min) and examined under a fluorescence microscope. The number of spermatozoa attached to the zona pellucida, and the quantity associated with each oocyte were quantified, providing evidence of both monospermy and polyspermy (Santos et al. 2023).

Presumptive zygotes and morulae were processed for scanning electron microscopy (SEM) to visualize their structure, following the protocol outlined by Vanroose et al. (2000), with minor modifications. They were fixed for 2 h in 2.5% glutaraldehyde and 0.1 M cacodylate buffer (pH 7.2) at 4°C, subsequently washed twice for 5 min in 2% cacodylate buffer, and post‐fixed in 1% osmium tetroxide for 2 h at 25°C. The structures were then washed in distilled water for 10 min, dehydrated through increasing concentrations of ethanol, and mounted on glass coverslips. After drying, the samples were gold‐coated. Finally, the ultrastructure of the cells was examined using a scanning electron microscope (TESCAN VEGA3; Tescan Analytics, Fuveau, Bouches‐du‐Rhône, France).

Reactive oxygen species (ROS) and mitochondrial membrane potential (ΔΨm) were quantified using 10 µm 2',7'‐dichlorodihydrofluorescein diacetate (H_2_DCFDA; Invitrogen, Carlsbad, CA, USA) and 500 nM MitoTracker Red® (Molecular Probes, Eugene, OR, USA), respectively, to evaluate the oxidative stress response. Both presumptive zygotes and morulae (fixed from D2 and D5, respectively) were incubated in the dark with the respective probes for 30 min at 38.5°C under 6.5% CO₂. Following incubation, the samples were placed on glass slides in microdroplets, and images were captured using a fluorescence microscope (Olympus BX51TF, Tokyo, Japan). Fluorescence intensity was quantified using ImageJ software (National Institutes of Health, Bethesda, Maryland, USA). The fluorescence values of each group were normalized to the mean of all groups to derive relative expression levels, which were expressed in arbitrary fluorescence units (AFU) (Santos et al. 2023).

### 2.7 Statistical Analysis

All data are expressed as the mean ± standard error and were analyzed using StatView 5.0 (SAS Institute Inc., Cary, NC, USA). Normality of all results was verified using the Shapiro‐Wilk test, and homoscedasticity was confirmed with Levene's test. ROS and ΔΨm were altered with arcsine and analyzed by variance analysis (ANOVA) followed by a Tukey test. All other data were compared using a chi‐squared test. Statistical significance was set at *p* < 0.05.

## 3 Results

All epididymal sperm samples exhibited a whitish color, with an average pH of 7.0, and a vigor score of 5.0 on a 0–5 scale. The average concentration following selection was 75 × 10^6^ sperm/mL. Additionally, a total of 18 ovaries were recovered from female red‐rumped agoutis, yielding 343 viable oocytes, corresponding to an average of 19.1 viable oocytes per ovary and 38.1 viable oocytes per female.

### 3.1 Sperm and Oocyte Evaluations

The sperm kinetics assessed through CASA analysis (Table S1) revealed that the sperm utilized for IVF exhibited optimal characteristics across all evaluated parameters, with an average total motility of 94.3% and progressive motility of 72.0%. Furthermore, the samples displayed a pronounced pattern of hyperactive‐like motility, as indicated by elevated VCL (187.9 µm/s) and ALH (5.8 µm/s) values. The quality of these samples was further confirmed by a membrane functionality rate of 90.0%, as determined by HOST. Furthermore, the analysis of membrane integrity and mitochondrial activity (Table S2) revealed that 70.0% of the sperm population exhibited an intact membrane and optimal mitochondrial function, while only 6.7% of the samples demonstrated impairments in both of these parameters.

The assessment of DNA damage levels in the sperm utilized for IVF revealed no abnormalities in the measured rates, with the following distribution: intact DNA (90.3% ± 1.2), low DNA damage (3.7% ± 1.2), moderate DNA damage (4.3% ± 1.2), and high DNA damage (1.7% ± 1.2%). Comparable results in terms of sample normality were observed in the morphological analysis (Table S3), where 93.3% of the sperm population was classified as normal. The abnormalities identified were evenly distributed across the head (2.0%), midpiece (3.0%), and tail (1.7%) regions.

**Figure 2:**
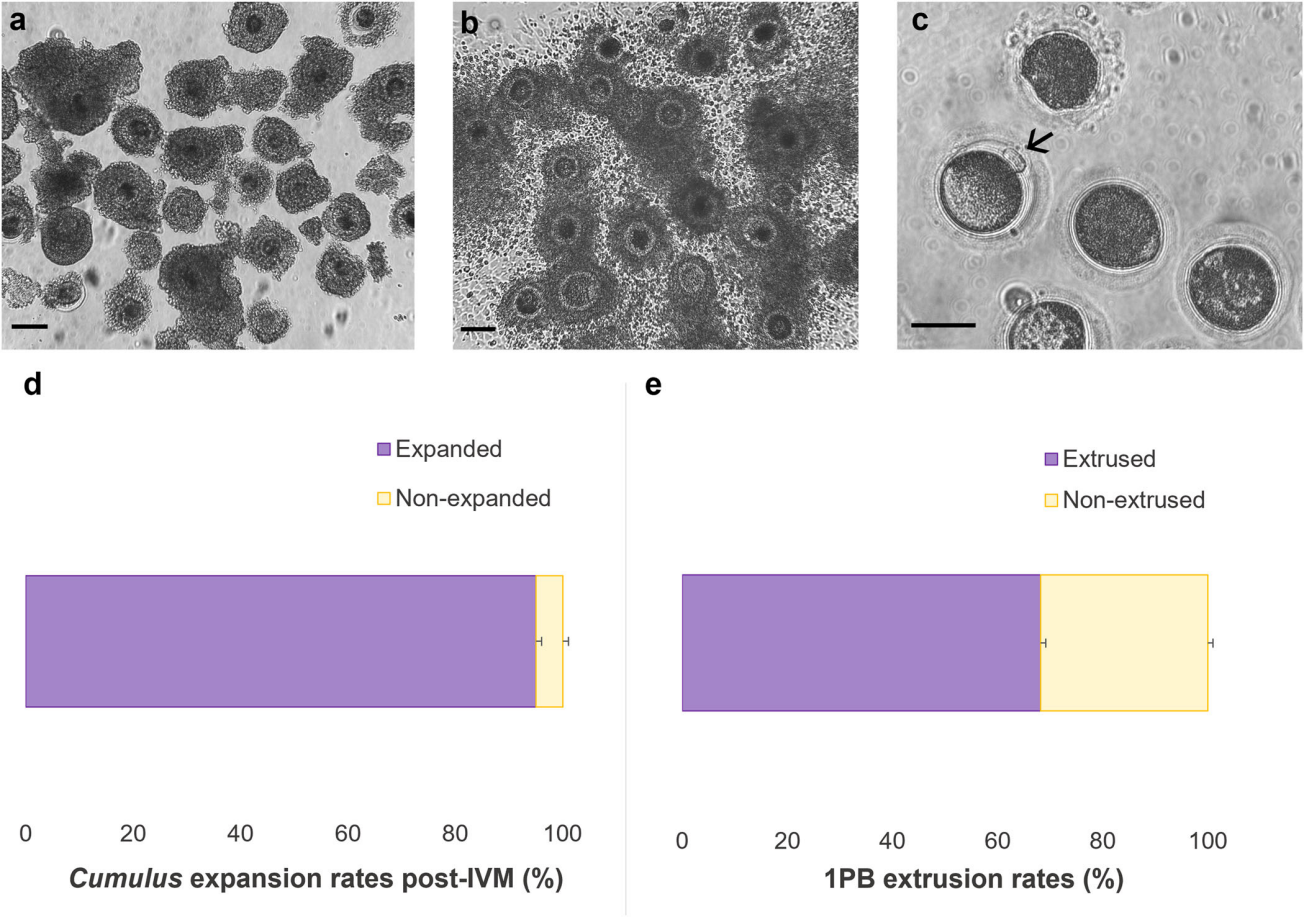
Efficiency of oocyte in vitro maturation of red‐rumped agouti. (a) Representative photomicrographs of immature red‐rumped agouti oocytes, (b) mature oocytes exhibiting *cumulus* cell expansion, and (c) nuclear‐matured oocytes with an extruded first polar body (1PB). (d) *Cumulus* cell expansion rate following IVM. (e) 1PB extrusion rate post‐IVM. Arrow indicates extruded 1PB. Scale bar: 100 μm (a,b) and 50 μm (c). Magnification: 10× (a, b) and 20× (c).

Following the IVM of red‐rumped agouti oocytes (Figure 2), *cumulus* cell expansion was observed at a rate of 95.0% ± 1.0 (326/343). Moreover, the extrusion of the 1PB occurred in approximately 68.2% ± 0.9 (234/343) of the oocytes.

### 3.2 Embryo Production and IVD

**Figure 3:**
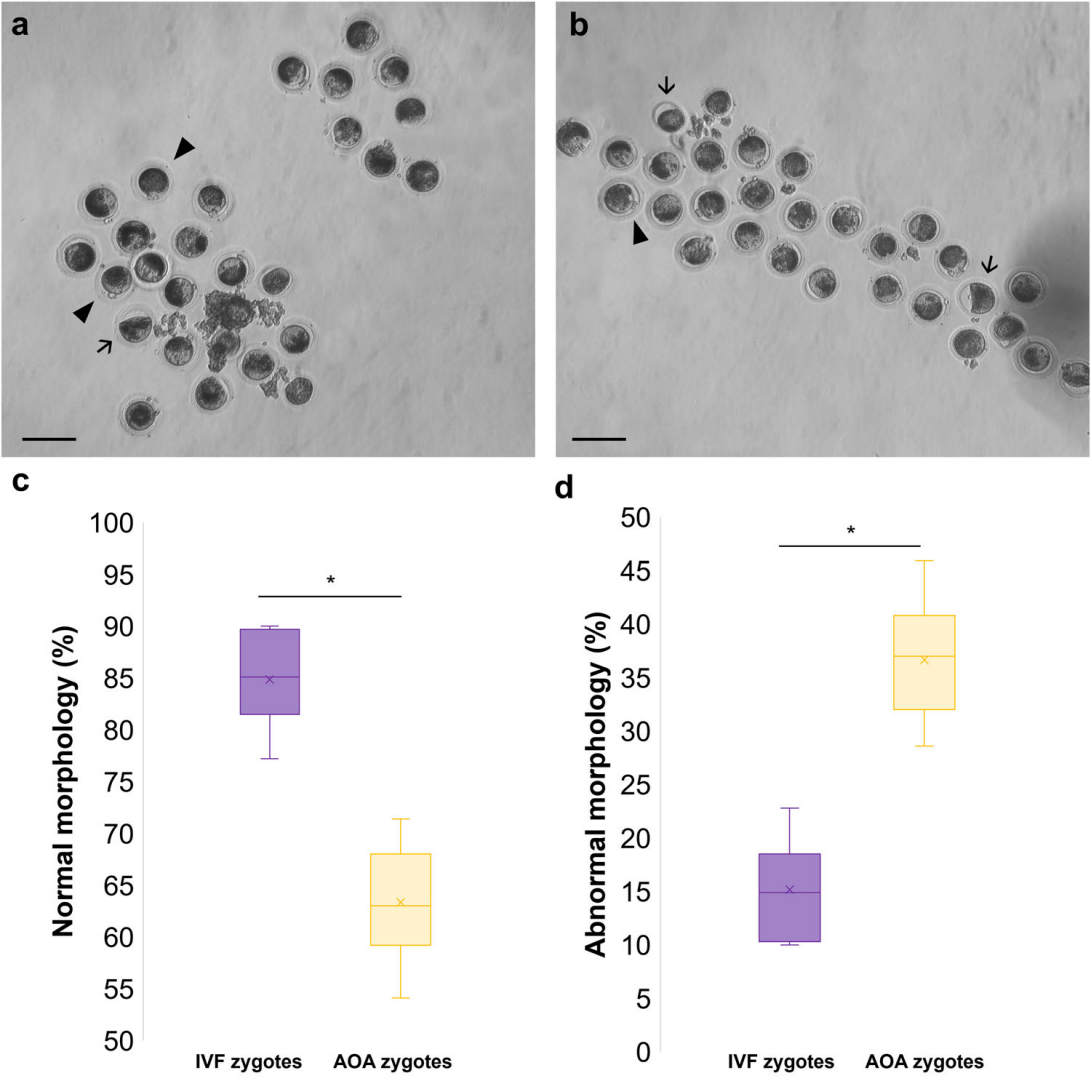
Efficiency of presumptive zygote production using different methods (in vitro fertilization vs. oocyte artificial activation) assessed through morphological analysis. (a) Representative image of presumptive zygotes derived from IVF and (b) AOA. (c) Proportion of zygotes exhibiting normal morphology and (d) those with abnormal morphology following IVF and AOA. The arrowhead denotes zygotes with normal morphology, while the arrow indicates those with abnormal morphology. Asterisks indicates statistically significant differences between groups (*p* < 0.05). Scale bar: 100 μm. Magnification: 10×.

**Figure 4:**
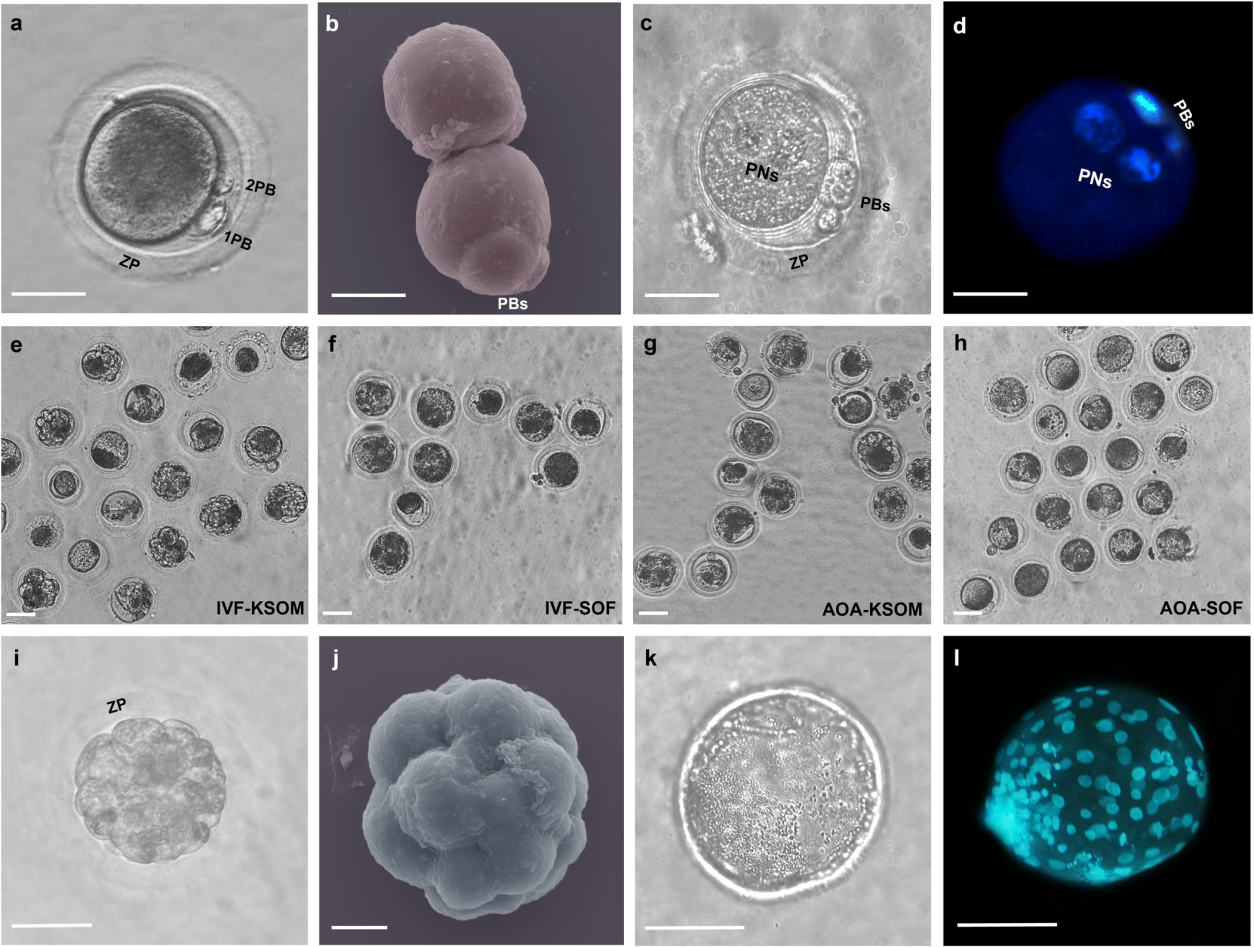
Embryo development kinetics following in vitro fertilization and oocyte artificial activation in the red‐rumped agouti. (a) Representative image of a presumptive zygote displaying the first and second polar bodies 6 h after IVF. (b) Colored scanning electron microscopy (SEM) image of a presumptive zygote without zona pellucida from the IVF‐KSOM group. (c) Visualization with an inverted microscope of female and male pronuclei 8 h after IVF. (d) H33342 staining of both pronuclei in an 8 h post‐IVF structure. (e–h) Representative images of IVD dishes on Day 5, illustrating cleavage in structures generated through IVF and AOA and cultured in KSOM or SOF media. (i) Morula produced via IVF on day 5 in KSOM medium, exhibiting well‐defined cellular cleavage. (j) Colored SEM image of a red‐rumped agouti morula on Day 5 after IVF. (k) Expanded blastocyst generated by the IVF‐KSOM group, visualized under an inverted microscope on Day 5 of IVD. (l) Expanded blastocyst from IVF of red‐rumped agouti stained with H33342 to identify blastomeres on day 5. ZP: zona pellucida. 1PB: first polar body. 2PB: second polar body. PBs: polar bodies. PNs: pronuclei. Scale bar: 10 μm (j), 20 μm (a–d), 50 μm (e–i; l), 60 μm (k). Magnification: 20× (e–h), 40× (a; c, d; i; k, l). SEM magnification: (j) 3.65 kx and (b) 2.27 kx.

The different embryo production methods had a significant impact on the morphology of presumptive zygotes (Figure 3a–d). IVF‐derived structures (Figure 4a–d) exhibited higher rates of normal morphological characteristics, an average of 84.5% ± 1.7 (147/174), whereas AOA‐derived structures showed only 63.3% ± 2.5 (107/169) (*p* < 0.05). No differences (*p* > 0.05) were observed in sperm‐oocyte interaction following IVF (Table 1), with monospermy rates exceeding 93% in both the SOF and KSOM groups.

**Table 1:** Red‐rumped agouti sperm interaction with oocyte after IVF when cultured in two different embryo media.

Groups	Sperm/oocyte (mean ± SE)	Sperm/zone (mean ± SE)	Monospermy (%)	Polyspermy (%)
KSOM	1.1 ± 0.1	52.0 ± 4.7	93.3 ± 6.7	6.7 ± 8.2
SOF	1.0 ± 0.0	62.3 ± 4.4	100.0 ± 0.0	0.0 ± 0.0

Mean ± standard error. *p* > 0.05.

Embryo kinetics analysis (Table 2) revealed significant differences between the techniques and media employed. On D2, both AOA‐KSOM (52.8%) and IVF‐KSOM (70.2%) yielded a higher number of cleaved structures compared to the groups cultured in SOF (37.0% and 42.5%) (*p* < 0.05). However, the cleavage rate of AOA‐KSOM was comparable to that of the SOF groups (*p* > 0.05).

**Table 2:** *In vitro* development of presumptive zygotes from red‐rumped agouti derived from both IVF and AOA cultured with different embryo culture media.

Groups	Percentage of cleavage/total zygotes at Day 2 (%)				Percentage of cleavage/total zygotes at Day 5 (%)				Morulae and blastocyst rate at Day 5 (%)			
	Total cleavage	2 cells	3‐7 cells	≥ 8 cells	Total cleavage	2 cells	3–7 cells	≥ 8 cells	Total morulae	Morulae/cleaved	Total blastocyst	Blastocyst/cleaved
IVF‐KSOM	70.2 ± 3.8 (52/74)^a^	7.7 ± 3.2 (4/52)^b^	28.8 ± 5.9 (15/52)^b^	63.5 ± 5.0 (33/52)^a^	78.4 ± 5.8 (58/74)^a^	6.9 ± 1.6 (4/58)^a^	5.2 ± 2.1 (3/58)^b^	87.9 ± 3.4 (51/58)^a^	50.0 ± 9.5 (37/74)^a^	63.8 ± 10.6 (37/58)^a^	1.3 ± 1.2 (1/74)^a^	1.7 ± 1.4 (1/58)^a^
IVF‐SOF	42.5 ± 4.1 (31/73)^b^	9.7 ± 2.2 (3/31)^ab^	74.2 ± 6.6 (23/31)^a^	16.1 ± 3.8 (5/31)^b^	45.2 ± 4.0 (33/73)^b^	15.2 ± 3.7 (5/33)^a^	30.3 ± 10.5 (10/33)^a^	54.5 ± 7.8 (18/33)^b^	10.9 ± 1.1 (8/73)^c^	24.2 ± 4.1 (8/33)^b^	0.0 ± 0.0 (0/73)^b^	0.0 ± 0.0 (0/33)^b^
AOA‐KSOM	52.8 ± 1.5 (28/53)^ab^	10.7 ± 0.4 (3/28)^ab^	32.1 ± 3.3 (9/28)^b^	57.1 ± 3.4 (16/28)^a^	58.5 ± 2.2 (31/53)^b^	3.2 ± 2.1 (1/31)^a^	32.3 ± 0.7 (10/31)^a^	64.5 ± 1.8 (20/31)^b^	30.2 ± 3.0 (16/53)^b^	51.6 ± 4.3 (16/31)^a^	0.0 ± 0.0 (0/53)^b^	0.0 ± 0.0 (0/31)^b^
AOA‐SOF	37.0 ± 2.3 (20/54)^b^	30.0 ± 4.0 (6/20)^a^	55.0 ± 4.9 (11/20)^ab^	15.0 ± 2.8 (3/20)^b^	40.7 ± 4.1 (22/54)^b^	13.6 ± 0.4 (3/22)^a^	45.5 ± 10.1 (10/22)^a^	40.9 ± 6.7 (9/22)^b^	9.3 ± 1.6 (5/54)^c^	22.7 ± 4.3 (5/22)^b^	0.0 ± 0.0 (0/54)^b^	0.0 ± 0.0 (0/22)^b^

Mean ± standard error. ^a,b,c^: values with different subscript letters in columns are significantly different (*p* < 0.05).

Regarding the cleavage rates on D2, presumptive zygotes cultured in SOF (AOA‐SOF and IVF‐SOF) exhibited a higher proportion of structures with fewer cells (30%), as compared to those cultured in KSOM (7.7%) (*p* < 0.05). In contrast, both AOA‐KSOM and IVF‐KSOM groups demonstrated a higher incidence of structures with ≥ 8 cells, with rates exceeding 55% (*p* < 0.05).

On D5, the IVF‐KSOM group exhibited the highest percentage of cleaved structures (78.4%) compared to all other groups (IVF‐SOF: 45.2%; AOA‐KSOM: 58.5%; AOA‐SOF: 40.7%) (*p* < 0.05, Figure 4e–h), underscoring the impact of both the technique and the IVD medium used. The IVF‐KSOM group also showed the highest percentage of structures with ≥ 8 cells (87.9%), when compared to IVF‐SOF (54.5%), AOA‐KSOM (64.5%) and AOA‐SOF (40.9%) (*p* < 0.05).

Furthermore, the total percentage of morulae on D5 was significantly higher (*p* < 0.05) in the IVF‐KSOM group (50.0%, Figure 4i, j) compared to the other groups (IVF‐SOF: 10.9%; AOA‐KSOM: 30.2%; AOA‐SOF: 9.3%). Additionally, AOA‐KSOM outperformed both IVF‐SOF and AOA‐SOF groups (*p* < 0.05), demonstrating the effectiveness of this culture medium for the species, regardless of the method employed. The superiority of KSOM was further evidenced by the morulae/cleaved ratio, where both AOA‐KSOM (51.6%) and IVF‐KSOM (63.8%) outperformed the SOF‐cultured groups (IVF‐SOF: 24.2%; AOA‐SOF: 22.7%) (*p* < 0.05). Notably, only the IVF‐KSOM group produced blastocysts (1.3%, Figure 4k, l), making it the most efficient among all evaluated groups (*p* < 0.05).

**Figure 5:**
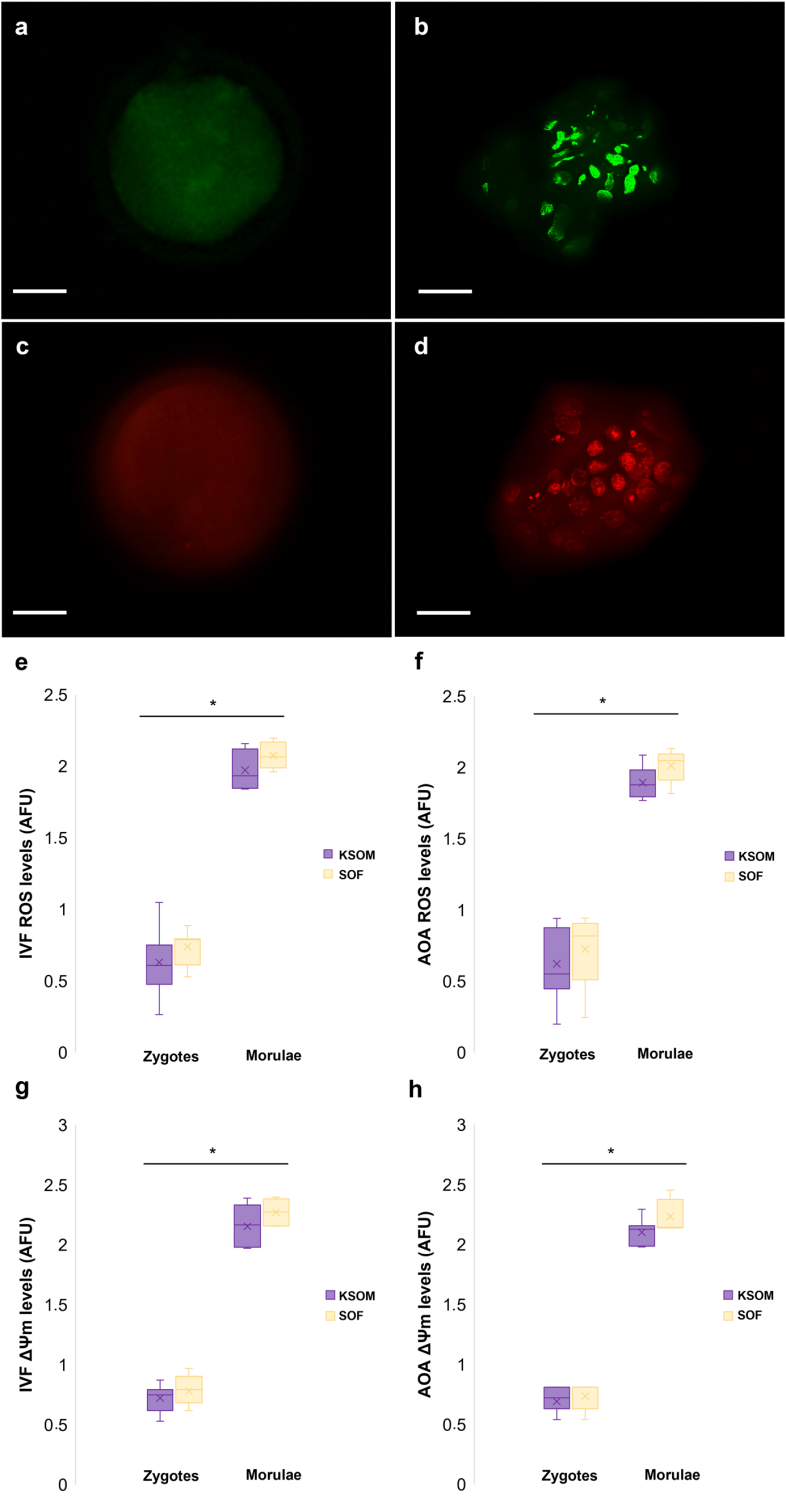
Effect of different embryo production methods (IVF vs. AOA) and embryo culture media (KSOM vs. SOF) on presumptive zygotes and morulae ROS production and mitochondrial membrane potential (ΔΨm). (a) Representative image of a zygote 6 h post‐IVF and (b) five‐day IVF morulae stained with the H_2_DCFDA fluorescent probe for assessing reactive oxygen species (ROS) levels. (c) Representative photomicrograph of a 6 h post‐AOA zygote and (d) five‐day AOA morulae labeled with the MitoTracker Red fluorescent probe to evaluate mitochondrial membrane potential (ΔΨm) levels. (e) ROS levels in IVF‐derived structures cultured in KSOM or SOF. (f) ROS levels in AOA‐derived structures cultured in KSOM or SOF. (g) ΔΨm levels in labeled IVF‐derived structures. (h) ΔΨm levels in labeled AOA‐derived structures. Asterisks indicate statistically significant differences between structure types (*p* < 0.05). Scale bar: 20 μm. Magnification: 40×.

Finally, no differences (*p* > 0.05) were observed among the groups (KSOM vs. SOF/IVF vs. AOA) in the assessment of oxidative stress (Figure 5). However, the mean levels of ROS were higher in the morulae (1.98 AFU ± 0.04) compared to the presumptive zygotes (0.68 AFU ± 0.04) (*p* < 0.05). The same results were observed in the mitochondrial membrane potential (ΔΨm), where the morulae presented a higher ΔΨm (2.18 AFU ± 0.04) compared to the presumptive zygotes (0.73 AFU ± 0.02) (*p* < 0.05).

## 4 Discussion

This study demonstrated that IVF using KSOM medium is currently the most effective approach for the in vitro production of red‐rumped agouti embryos. The optimal performance of IVF‐KSOM was evidenced by a lower incidence of zygotes with morphological abnormalities, higher cleavage rates up to D5, increased morula formation, and its distinction as the only group to surpass the IVD block in this species, successfully reaching the blastocyst stage for the first time. Despite the lower developmental rates observed with AOA, the use of the KSOM medium for the first time in this species improved the technique's efficiency.

Successful IVF in rodents relies on sperm samples exhibiting high motility, normal morphology, and intact DNA, as these parameters are strong predictors of male reproductive success in ARTs (Li and Lloyd 2020). We achieved exceptional results through optimizations in sperm selection and capacitation protocols for the red‐rumped agouti, conducted by our research team (Oliveira et al. 2025c), with total motility (TM) exceeding 94.0%. This represents a significant improvement compared to the findings of Castelo et al. (2023), who reported a TM of only 24.8% in the same species. Additionally, our study demonstrated an increase in progressive motility (PM), reaching 72.0%, whereas Dantas et al. (2022) observed a PM of just 13.7%. Moreover, the sperm samples used in IVF exhibited hyperactivated motility, a critical attribute that facilitates detachment from the isthmus reservoir and successful penetration of the surrounding oocyte envelopes (Giaccagli et al. 2021).

Furthermore, the normal sperm morphology observed in the samples used for IVF may have positively influenced the higher IVD rates. In mice, Kawai et al. (2006) have demonstrated that morphological abnormalities, such as tail breakage and defects in the midpiece, can reduce fertilization potential by up to 40%. The high proportion of intact DNA in our sperm samples further corroborates the efficiency of our IVF outcomes, as sperm DNA integrity has become one of the most extensively investigated biomarkers in reproductive biology. It is well established that DNA damage negatively impacts fertilization, embryonic development, implantation, and pregnancy success rates (Zhu et al. 2022).

The higher IVD outcomes in our study may be directly associated with proper oocyte maturation, as the cytoplasmic and nuclear modifications within the oocyte are essential for successful sperm interaction during fertilization (Fair and Lonergan 2023). Our results, together with those of Praxedes et al. (2023), demonstrated superior oocyte maturation outcomes when compared to the earlier findings of Ferraz et al. (2020). In our study, we achieved a *cumulus* cell expansion rate of 95.0% and a 1PB extrusion rate of 68.2%, while Praxedes et al. (2023) reported 100.0% *cumulus* expansion and 52.0% nuclear maturation in red‐rumped agouti (*Dasyprocta leporina*). These values contrast markedly with those obtained by Ferraz et al. (2020), who observed only 27.0% nuclear maturation after 24 h of IVM in black‐rumped agouti (*Dasyprocta prymnolopha*). The improved outcomes in both our study and that of Praxedes et al. (2023) can be attributed to the optimization of the IVM protocol, particularly through species‐specific adjustments in the concentrations of epidermal growth factor (EGF) and follicle‐stimulating hormone (FSH). These targeted modifications likely contributed to the higher maturation efficiency observed, reinforcing the importance of hormonal refinement in establishing effective ART protocols for neotropical rodents.

Upon comparing the outcomes of IVF and AOA in the development of red‐rumped agouti embryos, it was observed that, after 6 h of incubation, IVF yielded a higher number of morphologically normal zygotes. Although identifying embryos with the most significant implantation potential remains a persistent challenge in reproductive science, various strategies have been proposed in recent years to assess embryo viability, including the morphological assessment of zygotes (Stigliani et al. 2021). The poorer results observed in the AOA groups may be related to limitations inherent to parthenogenetic activation, particularly concerning genomic and cytoplasmic regulation. Hao et al. (2004) demonstrated that embryos generated by artificial activation often exhibit abnormal patterns of genomic imprinting and fail to undergo proper cytoplasmic reorganization during early development. Such disruptions can compromise crucial events like spindle assembly, organelle redistribution, and epigenetic reprogramming, ultimately reducing embryonic competence. These findings support the hypothesis that the absence of sperm‐derived factors during activation may impair the coordinated molecular processes necessary for normal morphological development.

Regardless of the IVD medium used, our observations revealed a favorable interaction between oocytes and sperm following IVF, with polyspermy occurring at a rate of approximately 6% or less. This is an important finding, as higher polyspermy rates, often surpassing 10% in IVF systems, can result in embryos that are unsuitable for transplantation and hinder their further development (Sun and Zhu 2023).

On D2 of IVD, the first cleavages revealed that embryos from both techniques cultured in KSOM medium displayed the highest frequencies of total cleavage and more than eight‐cells cleavage stage. This finding is noteworthy, as previous studies have shown that the timing of initial cleavage can be a crucial factor in identifying embryos with greater implantation potential (Lechniak et al. 2008). Additionally, transferring embryos from early cleaved zygotes has been associated with improved pregnancy and implantation rates compared to transferring embryos from zygotes that cleave later (Almagor et al. 2015).

The key factor contributing to the success of this study was the selection of KSOM as the IVD medium. Mammalian preimplantation embryos are highly sensitive to their surrounding environment, pivotal in influencing their developmental capacity by modulating key metabolic processes (Truong and Gardner 2017; Banrezes et al. 2025). Consequently, it is crucial to provide a culture medium tailored to the specific needs of each species. For example, in mice, Rostami et al. (2021) demonstrated that embryonic development is highly influenced by the composition of the in vitro culture (IVC) medium. In their study, the authors evaluated the effect of cyanocobalamin supplementation at different concentrations and found that the addition of 200 pM cyanocobalamin to the IVM, IVF, and culture media significantly improved developmental outcomes. Specifically, this concentration was associated with increased rates of 2‐cell stage embryos and blastocyst formation, alongside a marked reduction in the incidence of developmental arrest at the 2‐cell stage and in embryo degeneration.

KSOM is a well‐established culture medium for mouse embryos, which has notably advanced the field of IVEP for rodents by enabling the successful progression of embryos from the two‐cell stage blockage to blastocysts for the first time (Erbach et al. 1994). The superior performance of KSOM over SOF in red‐rumped agoutis can be attributed to the medium's unique composition, which includes a blend of essential amino acids, high glucose concentration (5.56 mmol/L), and bovine serum albumin (4 mg/mL). Research has demonstrated that these components are vital for supporting rodent embryo development, resulting in higher cleavage rates and increased blastocyst production across various mouse strains (Summers 2014).

Starting from D5, the IVF‐KSOM group exhibited superior cleavage rates and cell numbers compared to the AOA‐KSOM group, highlighting a distinct difference in embryonic development beyond the medium composition. One plausible explanation for the reduced developmental competence of AOA‐derived embryos is the absence of the paternal genome, which plays a critical role in normal embryogenesis. In embryos generated by IVF, both maternal and paternal gene expressions are essential for orchestrating proper developmental progression, as highlighted by Kharche and Birade (2013). The lack of paternal genetic contribution in parthenogenetic embryos may disrupt this balance, leading to impaired activation of key developmental pathways. Liu et al. (2002) suggest that this absence can prolong the cell cycle, which may explain the slower preimplantation development frequently observed in artificially activated embryos when compared to those generated by conventional fertilization. Edwards (2007) further supports this notion, reporting that delayed cleavage and reduced developmental kinetics are characteristic of embryos lacking paternal input. Additionally, as noted by Kharche and Birade (2013), a reduced DNA content in AOA embryos could compromise the regulation of gene expression networks, potentially increasing susceptibility to apoptosis or resulting in failure during critical stages of development. Together, these findings reinforce the hypothesis that the absence of the male genome is a central factor contributing to the suboptimal outcomes observed in AOA‐derived embryos.

To refine the AOA procedure for red‐rumped agouti, incorporating KSOM as the IVD medium demonstrated favorable outcomes, with a total cleavage rate of 58.5% and 30.2% morulae, exceeding the results obtained for other agouti species. In comparison, the activation in black‐rumped agouti, as outlined by Ferraz et al. (2020), achieved a cleavage rate of 63.6% on D2% and 15.1% morulae on D4. For red‐rumped agouti, Praxedes et al. (2023) reported a cleavage rate of 43.2% on D2% and 8.3% morulae production on D5, in the best‐performing experimental group. These variations can likely be attributed to the use of SOF medium in both studies, which could have affected the overall success of their IVEP. Our results support this hypothesis, as the AOA‐SOF group exhibited lower cleavage (37.0%), and morulae rates (9.3%) compared to the AOA‐KSOM group.

We observed an improvement in the method when comparing our results to those of Praxedes et al. (2023), using the same AOA protocol but with KSOM instead of SOF, reinforcing the superiority of KSOM as the medium for red‐rumped agouti. Despite this optimization, further adjustments are necessary in the pre‐IVD stages of artificial activation, particularly in fine‐tuning incubation times and identifying the most suitable protein activators or inhibitors to increase the success rate of in vitro embryo production.

This study represents the first successful report of IVF in red‐rumped agouti. Prior research by Ferraz et al. (2020) had already demonstrated the application of this reproductive biotechnology in black‐rumped agouti. They used a conventional bovine IVF protocol with a commercial medium, employing non‐capacitated epididymal sperm and a high sperm concentration of 25 × 10^6^ sperm/mL for oocyte co‐incubation. Their protocol involved a prolonged IVF duration of 15 h and used SOF as the IVD medium. Despite these efforts, the success rates were relatively low, with only 8% cleavage on D2% and 2.9% morulae on D5.

The better results observed in our study with red‐rumped agouti may be attributed to several key methodological differences. Specifically, we focused on optimizing the IVF protocol based on characteristics more closely aligned with rodent species. This included refined sperm selection (Oliveira et al. 2025a) and capacitation techniques (Oliveira et al. 2025c), the use of a lower sperm concentration, a proper IVF medium (Oliveira et al. 2025b), shorter IVF incubation period, and the selection of an IVD medium that is widely employed in rodent IVF protocols. We aimed to maximize the efficiency of each step by carefully evaluating and fine‐tuning each aspect of the process. As a result, this study marks the first successful production of a blastocyst via IVF in caviid rodents, demonstrating significant advancements in embryo development outcomes.

Finally, no differences were observed in ΔΨm levels or ROS production across the evaluated groups, indicating the stability of the culture environment regardless of the method or medium used. However, elevated levels were detected when comparing structures at the 6‐h incubation mark to morulae on day five. This increase may be associated with the transient rise in ΔΨm observed in late‐stage embryos, coinciding with embryonic genome activation, as previously reported in mouse IVF embryos (Acton 2004). Similarly, the increase in ROS production aligns with its essential role in regulating gamete function and embryonic development, where a higher cell count requires greater ROS levels than zygotes (Al‐Gubory et al. 2010).

In conclusion, the combination of IVF and KSOM medium has proven to be the most effective approach for IVEP in red‐rumped agoutis, promoting superior zygote morphology and improved embryonic development rates. Moreover, applying KSOM in AOA contributed to refining this method for this species. This study represents the first documented case of in vitro blastocyst production in red‐rumped agouti. It offers valuable insights into this species’ reproductive biology, aids in the refinement of protocols for closely related rodents and ultimately supports conservation efforts for the agoutis.

## Author Contributions

Conceptualization: L.R.M.O., A.F.P; Data curation: L.R.M.O., Alexsandra Fernandes Pereira; Investigation: L.R.M.O., L.V.C.A., Luana Grasiele Pereira Bezerra, Alexandre Rodrigues Silva, Alexsandra Fernandes Pereira; Methodology: L.R.M.O.; Visualization: L.R.M.O.; Writing – original draft: L.R.M.O.; Resources: M.F.O., Alexandre Rodrigues Silva, Alexsandra Fernandes Pereira; Writing – review & editing: Alexsandra Fernandes Pereira; Formal analysis: Alexsandra Fernandes Pereira; Funding acquisition: Alexsandra Fernandes Pereira; Project administration: Alexsandra Fernandes Pereira; Supervision: Alexsandra Fernandes Pereira.

## Conflicts of Interest

The authors declare no conflicts of interest.

## Supporting information


**Table 1:** Computer‐aided sperm analysis of red‐rumped agouti sperm used for IVF.

**Table 2:** Membrane integrity and mitochondrial activity rate in red‐rumped agouti sperm used for IVF.

**Table 3:** Sperm morphology analysis from red‐rumped agouti epididymal sperm for IVF.

## Data Availability

The data that support the findings of this study are available on request from the corresponding author (A.F. Pereira).
