# Supplementary material for: Effects of Embryo Production Method and Culture Medium on Embryonic Development in Red‐Rumped Agouti
Source: Cell Biol Int. 2025 Sep 11;49(12):1655–68. doi: 10.1002/cbin.70080 (PMC12605755; doi:10.1002/cbin.70080)
Supplement: Supplementary file 1 — Table 1: Computer‐aided sperm analysis of red‐rumped agouti sperm used for IVF. [file CBIN-49-1655-s001.docx]

**Supplementary Table 1.** Computer-aided sperm analysis of red-rumped agouti sperm used for IVF.

| **CASA parameters** | **(mean ± SE)** | |
| --- | --- | --- |
| Total motility (%) | | 94.3 ± 1.7 |
| Progressive motility (%) | | 72.0 ± 1.0 |
| VAP (µm/s) | | 160.3 ± 8.9 |
| VSL (µm/s) | | 138.5 ± 7.7 |
| VCL (µm/s) | | 187.9 ± 11.5 |
| ALH (µm/s) | | 5.8 ± 0.3 |
| BCF (Hz) | | 25.9 ± 1.4 |
| STR (%) | | 83.3 ± 0.3 |
| LIN (%) | | 71.0 ± 0.5 |
| Rapid (%) | | 88.7 ± 1.8 |
| Medium (%) | | 6.0 ± 0.5 |
| Slow (%) | | 0.0 ± 0.0 |
| Static (%) | | 5.3 ± 1.4 |

SE: standard error. VAP: velocity average pathway; VSL: velocity straight line; VCL: curvilinear velocity; ALH: amplitude of lateral head; BCF: beat cross frequency; STR: straightness; LIN: linearity. Sperm population: rapid, medium, slow, or static.
