# Supplementary material for: Effects of Embryo Production Method and Culture Medium on Embryonic Development in Red‐Rumped Agouti
Source: Cell Biol Int. 2025 Sep 11;49(12):1655–68. doi: 10.1002/cbin.70080 (PMC12605755; doi:10.1002/cbin.70080)
Supplement: Supplementary file 2 — Table 2: Membrane integrity and mitochondrial activity rate in red‐rumped agouti sperm used for IVF. [file CBIN-49-1655-s003.docx]

**Supplementary Table 2.** Membrane integrity and mitochondrial activity rate in red-rumped agouti sperm used for IVF.

| **IPM (+)**  **MF (+)** | **IPM (+)**  **MF (-)** | **IPM (-)**  **MF (+)** | **IPM (-)**  **MF (-)** | **IPM/TOTAL** | **MF/TOTAL** |
| --- | --- | --- | --- | --- | --- |
| 70.0 ± 1.1 | 22.7 ± 3.8 | 0.7 ± 0.6 | 6.7 ± 3.7 | 92.7 ± 4.3 | 70.7 ± 0.6 |

IPM: intact plasma membrane. MF: mitochondrial function. (+) presence. (-) absence. Mean ± standard error (in %).
