# Supplementary material for: Effects of Embryo Production Method and Culture Medium on Embryonic Development in Red‐Rumped Agouti
Source: Cell Biol Int. 2025 Sep 11;49(12):1655–68. doi: 10.1002/cbin.70080 (PMC12605755; doi:10.1002/cbin.70080)
Supplement: Supplementary file 3 — Table 3: Sperm morphology analysis from red‐rumped agouti epididymal sperm for IVF. [file CBIN-49-1655-s002.docx]

**Supplementary Table 3.** Sperm morphology analysis from red-rumped agouti epididymal sperm for IVF.

| **Sperm morphology** | **(% ± SE)** |
| --- | --- |
| Normal | 93.3 ± 2.7 |
| Abnormal | 6.7 ± 2.7 |
|  |  |
| **Abnormal morphology** | **(% ± SE)** |
| Head defects | 2.0 ± 1.1 |
| Middle piece defects | 3.0 ± 1.7 |
| Tail defects | 1.7 ± 0.8 |

SE: standard error.
